# Everybody's Page

**Published:** 1888-11-03

**Authors:** 


### Everybody's Page.

It is said that the white milky juice of the euphorbia helioscopia, commonly called the sun-spurge, is an admirable and painless cure for warts. In some parts of the country the peasants call it the wart weed.

For the information of those who like to collect old and curious weapons, it may be said that poisoned arrows are very dangerous treasures. It has been found that even at the end of eighty years they have not lost their toxic power.

Among the new diseases which have come in the train of our developing civilisation is electric sunburn, which affects those who work constantly under the electric light. The face becomes red, there is irritation about the eyes which leads to undesired weeping, and in about five days the skin peels off.

In the good old days, when there was some regard shown for human comfort but none for human health, it was customary to shut the doors of a church immediately after morning service on Sundays, in order that, thanks to the absence of ventilation, it might be warm in the evening.

As year after year passes by we are rising in the scale of being. Disease, crime, and death are diminishing, and although we know that to attain these benefits struggles are required and sacrifices are entailed, we know also that it is within our power individually and collectively to make these struggles less severe and these sacrifices less numerous for ourselves and for those who come after us.—*Alex. James, M.D.*

The bath for a child during the first week of its life should be only a little below blood-heat, and should be given in a room in which there is a sufficiently good fire to permit of the bathing being conducted at a distance from it. After a few weeks the morning bath may be made a little cooler, but the evening bath should not be lowered in temperature till the child is three months old, when the bath may be reduced to seventy or eighty degrees.

The workers in the quicksilver mines of Almaden, in Spain, are subject to chronic pneumonia, which is rendered more dangerous by anemia, and a gradual development of mercurial poisoning, which causes a rapid deterioration of the substance of the nervous centres. During the five years 1883-87, the average death rate of the mining population was 16 per cent. A large proportion of the miners are children under sixteen, and these succumb most readily to the unhealthy conditions under which they work.

It must be grievous to those who pride themselves on their blue blood to know how brief, after all, is the life of an old family. Dr. Gustave Lagneau, who has recently been investigating the question, shows that all aristocracies tend to become smaller unless reinforced by the constant introduction of new—and presumably less azure—blood. The oldest families have rarely lasted more than three hundred years, and many proud houses have become extinct within a century of their coming prominently before the world.

Some interesting details of the food loved by the Carthaginians of old are given in Flaubert's novel, "Salammbô." Among their festal dishes were snails (dressed with cumin), roasted antelopes (garnished with their horns), peacocks in their plumage, legs of camels and buffaloes, hedgehogs, fried grasshoppers, and preserved dormice. Every dish was plentifully flavoured with truffles and assafœtida, and the greatest luxury of all was a species of red-haired and plump little dog, a dainty which won for the Carthaginians the loathing of their neighbours.

The praise of "the roast beef of old England, the old English roast beef," is chanted by M. Jules Simon in this week's *Journal d'Hygiene.* He says that the Paris workman spends a great deal on his food, and withal is very ill-fed. He drinks acid wines and eats bad *charcuterie,* loads his stomach with heavy and unhealthy food, while for the same money an Englishman gets good beer and what he defines as "un roast beef succulent." Let us, mingling our tongues after M. Simon's example, sing "Vive le roast beef, à bas les kickshaws."

One of the trials of the average man is the acquaintance who declares that he can rise with the lark without going to bed with the lamb. No absolute rule can be laid down as to the time we should spend in sleep. Some famous individuals, such as Frederick the Great, John Hunter the great surgeon, and Sir George Elliot, the defender of Gibraltar, have got on very well with only four hours' sleep per diem. While looking on these personages and others who emulate them with a respectful envy, it is well to admit that for most of us eight hours' sleep is necessary and nine not too much.

Milk is one of the best medicines in the pharmacopœia, or out of it. Beef tea rarely contains more than a fourth of the nutriment of milk, and raw beef juice is only equal to it in the nutritive scale. In diseases of the stomach, milk is invaluable, and if the patient finds it too heavy, lime, soda, or potass water may be added, or he may take skim-milk, which is deprived of the fat, or whey, which has no curd and is very easily absorbed. In consumption, milk is an essential article of diet, and in Bright's disease it forms an important part of the treatment. In fact, we may almost say with the country minister, who took it as an adjunct to chicken gril, "Milk's guid wi' a' things."

Is it dignified, is it permissible, for a medical man to visit his patients on a bicycle or tricycle is a question asked by "A Junior Partner" in the *British Medical Journal.* Why not? Dignity dwelleth not in frock-coats, nor does competence inevitably drive in a carriage, and patients, as a rule, are quick enough to apprehend these things. If a man knows his work he may go to it fearlessly by any means of locomotion, from "shanks' naggie" upward. And the iron steed has many things to recommend it. It is cheaper than a horse to begin with, and, on the whole, more trustworthy. It doesn't bolt, even at the sight of a road engine, it doesn't fall ill, it never gets fatigued, and all it asks is a good wash and a little oil after a hard day's work. It is a pity that more medical men do not make a practical use of cycling.

While a great deal has been said, and justly said with regard to the insanitary houses of the working classes, it should be remembered that they are often inhabited by preference. The working man who wishes to live in a better house, must often choose between it and his pipe, his beer, or his ample—too ample—Sunday dinner. His wife's best clothes must be sacrificed too, or else, if she is a proud woman, and likes the thought of owning a house of four rooms, she thinks she is fulfilling the duty of the ideal wife and mother if she lets three of them to lodgers, and lives with all her family in the kitchen. Even when new, clean, and wholesome dwellings are provided at a low rent, the British workman refuses to inhabit them. They are too clean, and they are to be kept clean, which is a distressing and insulting necessity, for if there is exaggeration, there is not absolute untruth in Mr. Anstey's picture of the coster whose "pride was cruel hurt, to think them swells could ha' gone so far as to rob a poor man of his dirt."

